# Cardiorespiratory fitness is associated with sickness absence and work ability

**DOI:** 10.1093/occmed/kqac070

**Published:** 2022-08-12

**Authors:** P Kolu, J Raitanen, H Sievänen, K Tokola, H Vähä-Ypyä, E Nieminen, T Vasankari

**Affiliations:** UKK Institute for Health Promotion Research, Tampere, Finland; UKK Institute for Health Promotion Research, Tampere, Finland; Faculty of Social Sciences (Health Sciences) of Tampere University, Tampere, Finland; UKK Institute for Health Promotion Research, Tampere, Finland; UKK Institute for Health Promotion Research, Tampere, Finland; UKK Institute for Health Promotion Research, Tampere, Finland; Management of Human Resources, City of Nokia, Nokia, Finland; UKK Institute for Health Promotion Research, Tampere, Finland; Faculty of Medicine and Health Technology of Tampere University, Tampere, Finland

## Abstract

**Background:**

Physical activity may sustain the physical aspect of work ability despite health problems such as musculoskeletal disorders and anxiety, which are the most prevalent work-related health problem in Europe.

**Aims:**

To evaluate the association of Finnish municipal workers’ accelerometer-measured physical activity, sedentary behaviour, and cardiorespiratory and muscular fitness with their sickness absence levels, perceived work ability and health-related quality of life.

**Methods:**

In connection with a randomized controlled trial recruiting 185 municipal workers, the authors performed baseline data analysis utilizing quantile regression to examine relationships between the outcome variables (all-cause sickness absence for 6 months, perceived work ability and health-related quality of life) and cardiorespiratory fitness, muscular fitness, and physical activity, and sedentary behaviour. All results were adjusted for age, sex and education level.

**Results:**

The median duration of all-cause sickness absence over the preceding 6 months was lowest among participants with high cardiorespiratory fitness relative to the lowest tertile (2.0 versus 6.0 days; *P* < 0.05), and the highest perceived work ability was found among those with high or moderate cardiorespiratory fitness as compared to the lowest tertile (8.0 versus 7.0; *P* < 0.001). Moderate-to-vigorous physical activity correlated positively with the physical component of health-related quality of life (*P <* 0.01) and with a high cardiorespiratory-fitness level (*P <* 0.05).

**Conclusions:**

High cardiorespiratory fitness was associated with decreased all-cause sickness absence days and improved work ability among municipal workers.

## Teaser

In Europe, musculoskeletal disorders, such as lower-back pain, constitute the most prevalent work-related health problem, irrespective of the type of work. According to a study of employees of a Finnish municipality, high cardiorespiratory fitness was associated with decreased all-cause sickness absence days and improved work ability among municipal workers. Therefore, the importance of good cardiorespiratory fitness should be further emphasized.

**Key learning points**

What is already known about this subject:

- Physical activity may sustain the physical aspect of work ability despite health problems such as musculoskeletal disorders and anxiety, which are the most prevalent work-related health problems in Europe.
- Studies have shown regular physical activity reduces the days of sickness absence and improves health-related quality of life but those findings have so far relied on self-reported activity levels.

What this study adds:

- High cardiorespiratory fitness is associated with lower levels of all-cause sickness absence and with higher perceived work ability among municipal workers.

What impact this may have on practice or policy:

- Reduced cardiorespiratory fitness is associated with substantial financial losses for employers and society both, arising from lost productivity—manifested as, for example, sickness absence.
- The importance of good cardiorespiratory fitness should be further emphasized.

## Introduction

Work ability is a combination of the employee’s physical and mental resources, work conditions and the policies affecting the work community [1]. In Europe, musculoskeletal disorders, such as lower-back pain, constitute the most prevalent work-related health problem (at 60% of all reported cases), irrespective of the type of work [2]. These are followed by stress, depression and anxiety, accounting for 16% [2]. In Finland, mental disorders (F00–F99 in the classification of diseases, version 10) are the main reason for extended sickness absences: they account for 34% of sickness absences longer than 10 working days, leading to nearly 5.0 million days of absence in 2020 alone [3]. Musculoskeletal disorders are the second most common cause accounting for 26% of at least 10 days long sickness absences, resulting in over 3.8 million sickness absence days in 2020 [3].

Physical activity may sustain the physical aspect of work ability [1] despite such health problems as musculoskeletal disorders [4] and anxiety [5]. Also, regular physical activity could improve quality of life through better physical functioning and health [5]. Consequently, physical activity may hold the potential to decrease the amount of sickness absence [6], which today constitutes an economically significant burden on employers and society at large [7]. Moreover, high cardiorespiratory fitness could play an especially significant role in reducing the duration and frequency of these absences [8]. Finally, there may be a link also between high cardiorespiratory and muscular fitness, coupled with engaging in the recommended amount of physical activity (at least 150 minutes of moderate- or 75 minutes of vigorous-intensity physical activity each week) [5], and lower total healthcare costs, at least among nursing personnel with recurrent low-back pain [9].

Prior studies point in this direction given the observed positive associations of higher self-reported physical activity with work ability, evaluated via a single item [10] and with work ability index [11]. Cardiorespiratory fitness too has shown an association with the latter index [12]. Consequently, there is some evidence that work ability predicts future sickness absences and the costs of occupational health care [13].

Likewise, studies have shown that regular physical activity reduces days of sickness absence and improves health-related quality of life (HRQoL) [5,6]. According to Kettunen *et al.* [14,15], having both high levels of leisure-time physical activity and good muscular and cardiorespiratory fitness was associated with stronger mental resources and low stress in normal-weight young men. Those findings relied on self-reported activity levels, however, whereas the study reported upon here went further. We aimed to evaluate the relationships of accelerometer-measured physical activity and sedentary behaviour, cardiorespiratory fitness and muscular fitness with sickness absence levels, perceived work ability and health-related quality of life among municipal workers.

## Methods

This cross-sectional study (*n* = 185) represents the baseline data analysis of a 6-month two-arm randomized controlled trial (clinical trial registration NCT03854201), the intervention arm of which was designed to improve municipal employees’ work ability by providing personalized exercise counselling that guided and motivated them to set and reach personal physical-activity goals. All data used and analysed in this study were collected before randomization. Data collection, except for the physical and cardiorespiratory-fitness tests and sickness absence, was conducted online using the Webropol Survey & Reporting tool (https://www.webropol.co.uk/).

Participants were recruited via the intranet of the city of Nokia (a town in southern Finland with about 34 000 inhabitants), with the municipality’s workers receiving a briefing in December 2018. After self-enrolment, the research assistant in the UKK Institute interviewed by phone all participants who were eligible and willing to participate. The final selection of participants was made by telephone interview. Participation was ruled out because of (i) health restrictions preventing moderate-intensity leisure-time physical activity, (ii) lack of trust in still holding one’s current work position after 2 years [16], (iii) training for a new work position or undergoing informal re-education, (iv) participation in any rehabilitation intervention or current plans to do so or (v) being pregnant.

Participants’ baseline tests were implemented in January–June 2019. The primary target population of the study consisted of practical nurses (*n* = 58), cleaning-service workers (*n* = 16), kitchen personnel (*n* = 12) and janitorial service workers (*n* = 2), as these occupations represented the ones with known challenges in sickness absence and musculoskeletal problems [2]. However, the scope needed to be widened to other municipal workers because the initial number of volunteers proved insufficient. The other included participants were teachers (*n* = 25), registered nurses (*n* = 21), other healthcare workers (*n* = 9), office personnel (*n* = 21), childcare workers (*n* = 4), various others (*n* = 12) and people who did not specify their occupation (*n* = 5). Also, the initial inclusion criteria specified at least 2 years before retirement age and self-assessed work ability below 8 on a 0–10 numeric rating scale [16]. However, again for a sufficiently large number of suitable participants, the latter criterion was loosened to 9. Each participant’s work ability was categorized as poor (scores of 0–5 points), moderate (6–7) or good/excellent (8–10) [16]. The Regional Ethics Committee for the Tampere University Hospital expert-responsibility area approved the study (ETL code R18184), and all participants provided their written informed consent to participate at the first study appointment.

Participants’ height and weight were measured before a physical fitness test, for which they were requested to avoid heavy meals for 2–3 h. The outcome variables of the present study were sickness absence, employee-perceived work ability and HRQoL.

All-cause sickness absences during the preceding 6 months were retrospectively obtained from the employer’s register data for each participant. To avoid overestimation, we included only weekdays in our calculations. Daily costs per sickness absence (in Euros) were expressed as price levels of the year 2019, when the study was implemented [17]. To factor in other related expenses, the daily cost of sickness absence was €183, which was obtained by multiplying the median national monthly salary of municipal workers [17] by 1.3 [18]; any costs caused by substitutes were not included. The cost calculations assumed 21 workdays a month.

Work ability was assessed online via a single question related to current work ability compared to the highest work ability ever: ‘Assume that your work ability at its best has a value of 10 points. How many points would you give your current work ability?’ This is the first question in the work ability index [19]. The answer denotes an integer rating on a scale from 0 (for inability to work at all) to 10 (denoting the respondent’s work ability at its best). This approach to the employee’s self-assessment of the current work ability entails an explicit comparison with the respondent’s lifetime-best rating.

The HRQoL was also assessed online, and the evaluation was based on the Research and Development 36-item health survey (RAND-36) electronic questionnaire [20], derived from the original SF-36 questionnaire [21]. The RAND-36 score covers eight dimensions of HRQoL: physical functioning, physical role limitation, bodily pain, general health, vitality, social functioning, emotional role limitation and mental health [20]. The index is expressed as a single number, from 0 (denoting the worst possible state of health) to 1 (representing the best possible state). Decreased work ability influences both physical and mental aspects of health as measured via the RAND-36 instrument [1].

The intensity and amount of weekly physical activity and sedentary behaviour were assessed using a triaxial accelerometer worn on the hip at least 4 days at a minimum 10 h a day [22]. The device was attached to an elastic belt at the right hip [23]. A hip-worn accelerometer enables differentiating not only between physical activity and sedentary behaviour but also between standing and a sedentary state—i.e., sitting or lying down [23]. The parameters describing the physical activity and sedentary behaviour were derived from raw accelerometer data via a 1-min exponential moving average of 6-s epoch data [23]. Intensity of physical activity is expressed as metabolic equivalents (METs), which expresses the level of the body’s oxygen consumption as multiples of the resting rate [24].

Under current recommendations, adults should have, at least, 150 min of moderate-intensity physical activity a week, or at least 75 min of vigorous-intensity aerobic physical activity a week, or a combination of them [5], with the suggested approach being sessions of 10-min or greater duration at least three times per week. Cardiorespiratory fitness was measured with 6-min walk test (6MWT) [25], carried out on a 15-m indoor track. The 6MWT was performed without a warm-up period but it was done after a modified push-up test, which may have served as a warm-up [26]. The 6MWT predicted the maximal oxygen uptake using walking distance, heart rate, body mass index (BMI), age and sex as predictors with accuracy of about one MET [25]. The modified push-up test evaluated the upper body’s ability to stabilize the trunk to gauge muscular fitness [26].

The analysis considered the background characteristics in terms of the mean and standard deviation (SD) or frequency and proportion. Cardiorespiratory and muscular fitness were broken down into tertiles for the statistical comparisons. The attrition rate of the study was the following: 182/185 participants filled an electronic questionnaire, 173 participants wore accelerometer at least 4 days at a minimum 10 h a day, 169 participants participated in muscular fitness test and 169 participants participated in 6MWT. An independent-samples *t*-test and ANOVA quantified the group differences in cases of normal-distribution baseline variables, with the Mann–Whitney *U*-test and Kruskal–Wallis techniques being employed for the other variables. The associations between categorical variables (e.g. education level) and groups were examined via Pearson’s chi-squared test. For adjustments of age, sex and education level and to address skewed outcome distributions, quantile (median) regression aided in examining the relationship between group membership and each of sickness absence days, work ability and HRQoL variables. In order to test whether meeting the physical activity recommendations, cardiorespiratory fitness and other explanatory variables were depending on the work demand (i.e., blue collar or office workers), an interaction term between explanatory variables and the work demand was additionally fitted to the models. The results were considered statistically significant if *P* < 0.05. For all analyses, the researchers employed Stata, version 15.1 (StataCorp, College Station, TX, USA).

## Results

The accelerometer data indicate that roughly a fifth of the study’s 185 participants (21%) met the physical-activity recommendations (see Table 1). The table also shows that those employees meeting the recommendations had a lower mean weight (71.2 versus 83.5 kg; *P* < 0.001) and BMI (26.4 versus 30.1 kg/m^2^; *P* < 0.001) than the inactive ones. Lower weight and BMI showed a significant association (*P* < 0.001) with good cardiorespiratory fitness (see Table 1).

**Table 1.** Background characteristics (mean and SD or frequency and proportion)

	All	Meeting physical activity recommendations			Cardiorespiratory fitness (based on the results of 6-min walk test)			
		Yes	No	*P*	High	Moderate	Low	*P*
*n*	185	38	135		42	44	83	
Age, mean (SD)	50.6 (8.7)	52.4 (8.2)	50.0 (8.8)	NS^a^	51.3 (8.7)	51.3 (9.1)	49.9 (8.3)	NS^b^
Weight (kg), mean (SD)	80.6 (17.2)	71.2 (11.1)	83.5 (17.8)	***^a^	64.4 (7.9)	74.4 (7.6)	92.0 (16.0)	***^b^
BMI (kg/m^2^), mean (SD)	29.2 (5.9)	26.4 (4.0)	30.1 (6.2)	***^a^	23.8 (2.5)	27.0 (3.0)	33.1 (5.5)	***^b^
Sex, *n* (%)				NS^c^				NS^c^
Women	168 (93)	35 (92)	125 (94)		38 (90)	50 (91)	79 (96)	
Men	12 (7)	3 (8)	8 (6)		4 (10)	4 (9)	3 (4)	
Education level, *n* (%)				NS^c^				NS^c^
Low	84 (47)	19 (51)	62 (46)		18 (43)	19 (44)	40 (48)	
Medium	40 (22)	7 (19)	32 (24)		13 (31)	7 (16)	18 (22)	
High	57 (31)	11 (30)	41 (30)		11 (26)	17 (40)	25 (30)	
Self-evaluated work ability (scale 0–10), mean (SD)	7.2 (1.3)	7.5 (0.8)	7.1 (1.4)	NS^d^	7.6 (1.0)	7.3 (1.3)	7.0 (1.4)	*^e^
Number of sickness absence days^f^, mean (SD)	6.8 (9.1)	7.1 (11.1)	6.8 (8.6)	NS^d^	7.6 (12.9)	4.4 (6.8)	7.3 (6.9)	*^e^
Physical quality of life index (scale 0–100), mean (SD)	73.4 (14.6)	79.0 (11.9)	71.4 (15.1)	**^d^	79.0 (12.2)	77.0 (14.0)	69.5 (14.6)	**^e^
Mental quality of life index (scale 0–100), mean (SD)	73.0 (16.6)	79.0 (13.7)	71.3 (16.9)	**^d^	77.7 (13.1)	73.6 (15.9)	70.3 (18.3)	NS^e^
SF index (scale 0–1), mean (SD)	0.72 (0.10)	0.75 (0.09)	0.70 (0.10)	**^a^	0.74 (0.09)	0.72 (0.10)	0.71 (0.10)	NS^b^

NS = non-significant; SF index = the six-dimensional health state short form (SF-6D) index.

^a^Independent-samples *t*-test.

^b^One-way ANOVA (the difference between three cardiorespiratory fitness groups).

^c^Chi-squared test (the association between two categorical variables).

^d^Mann–Whitney *U*-test.

^e^Kruskal–Wallis test (the difference between three cardiorespiratory fitness groups).

^f^During past 6 months.

*P < 0.05; **P < 0.01; ***P < 0.001.

**Figure 1.**
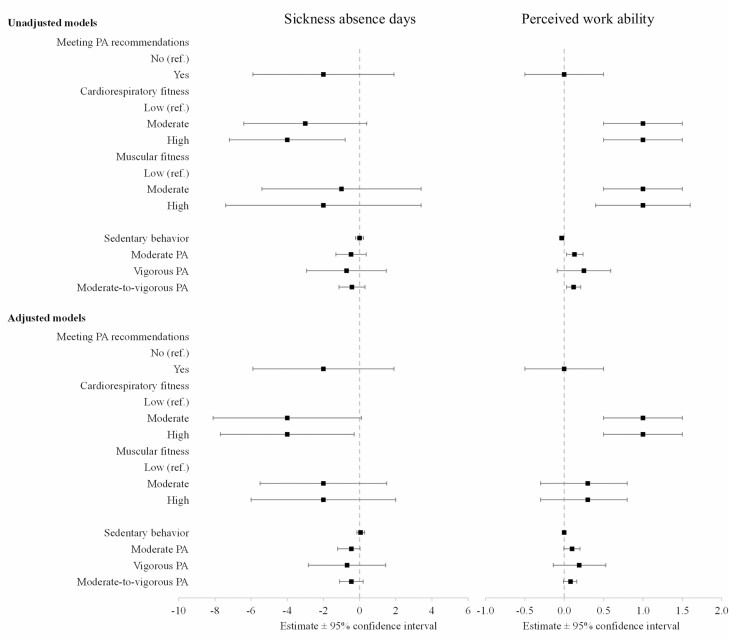
The parameter estimates for sickness absence days and perceived work ability and the quantile (median) regression models, with their 95% confidence intervals (CIs).

**Figure 2.**
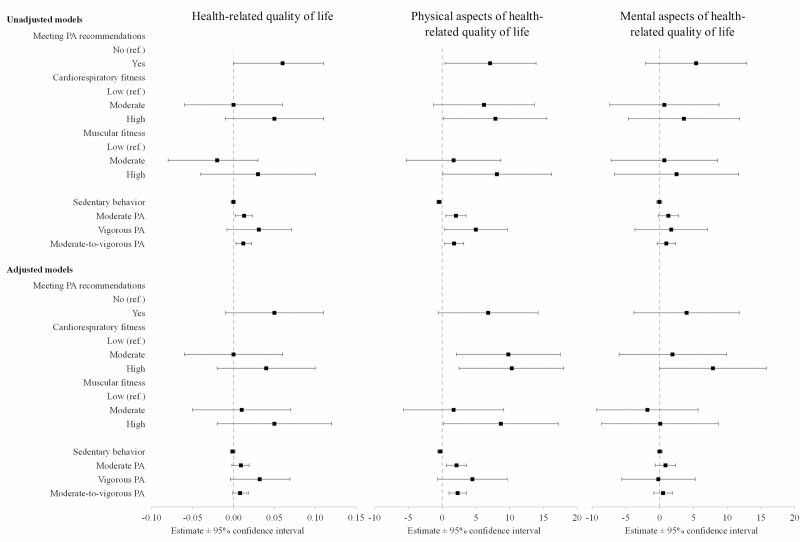
The parameter estimates for health-related quality of life (on a 0–10 scale) and for physical and mental aspects of health-related quality of life (on a 0–100 scale) and the quantile (median) regression models, with their 95% confidence intervals (CIs).

The median for all-cause sickness absence over the 6 months was lowest among employees exhibiting high cardiorespiratory fitness and highest for participants in the lowest tertile (2 versus 6; *P* < 0.05) (see Table 2 and Figure 1). Representing median sickness absence levels and associated costs, workers with high cardiorespiratory fitness created 67% lower sickness absence costs than the lowest tertile’s (€366 versus €1098; *P* < 0.05) during the preceding 6 months. No significant associations were seen between sickness absence and meeting of physical-activity recommendations, muscular fitness or level of physical activity (see Figures 1 and 2).

**Table 2.** Median (med) with range and mean with SD of sickness absence days and costs and perceived work ability (on a 0–10 scale)

	Sickness absence				Perceived work ability	
		Days	Costs			
	*n*	Med (min.; max.)	Med (min.; max.)	Mean (SD)	*n*	Med (min.; max.)
All	105	3.0 (0; 48)	€549 (€0; €8784)	€1258 (€1677)	173	7.0 (1.0; 9.0)
Meeting physical-activity recommendations						
No	82	4.0 (0; 48)	€732 (€0; €8784)	€1245 (€1575)	135	7.0 (1.0; 9.0)
Yes	23	2.0 (0; 44)	€366 (€0; €8052)	€1305 (€2037)	38	7.0 (6.0; 9.0)
Cardiorespiratory fitness						
Low	50	6.0 (0; 29)	€1098 (€0; €5307)	€1329 (€1262)	83	7.0 (1.0; 9.0)
Moderate	24	2.5 (0; 30)	€476 (€0; €8784)	€808 (€1252)	44	8.0 (3.0; 9.0)
High	31	2.0 (0; 48)	€366 (€0; €8784)	€1387 (€2356)	42	8.0 (3.0; 9.0)
Muscular fitness						
Low	55	4.0 (0; 48)	€732 (€0; €8784)	€1344 (€1693)	86	7.0 (1.0; 9.0)
Moderate	28	2.5 (0; 36)	€476 (€0; €6588)	€824 (€1327)	44	8.0 (3.0; 9.0)
High	16	2.0 (0; 44)	€366 (€0; €8052)	€1304 (€2090)	29	8.0 (6.0; 9.0)

Median work ability was highest among employees with high or moderate cardiorespiratory fitness as compared to the lowest tertile (8.0 versus 7.0; *P* < 0.001), but the results showed no significant differences in work ability in relation to meeting the physical activity recommendations or having higher muscular fitness or physical activity (see Table 2 and Figure 1).

HRQoL did not exhibit a significant relationship with meeting the physical activity recommendations or with having high muscular or cardiorespiratory fitness (see Table 3 and Figure 2). Meanwhile, moderate-to-vigorous physical activity did show a significant relationship with the physical HRQoL component (*P* < 0.01), as did a high level of cardiorespiratory fitness (*P* < 0.05). In addition, high cardiorespiratory fitness showed marginally significant (*P* < 0.05) relationship with the mental component of HRQoL (see Table 3 and Figure 2).

**Table 3.** Median (med) and range for health-related quality of life (on a 0–1 scale) and for physical and mental aspects of health-related quality of life (on a 0–100 scale)

	HRQoL		Physical aspect of HRQoL		Mental aspect of HRQoL	
	*n*	Med (min.; max.)	*n*	Med (min.; max.)	*n*	Med (min.; max.)
All	165	0.70 (0.47; 0.93)	173	75.2 (31.7; 98.8)	170	76.3 (25.0; 100.0)
Meeting physical-activity recommendations						
No	127	0.70 (0.47; 0.93)	135	73.3 (31.7; 98.8)	135	74.8 (25.0; 98.6)
Yes	38	0.76 (0.61; 0.92)	38	80.8 (46.0; 95.5)	38	80.6 (36.8; 100.0)
Cardiorespiratory fitness						
Low	80	0.70 (0.47; 0.85)	88	73.1 (31.7; 90.7)	81	75.0 (26.4; 98.6)
Moderate	41	0.70 (0.54; 0.92)	44	80.5 (48.3; 98.8)	43	75.7 (29.6; 100.0)
High	40	0.75 (0.59; 0.93)	42	81.1 (36.7; 95.5)	42	78.9 (25.0; 97.1)
Muscular fitness						
Low	82	0.73 (0.51; 0.93)	86	74.4 (37.6; 95.5)	85	75.7 (25.0; 98.6)
Moderate	42	0.70 (0.47; 0.89)	44	75.5 (34.3; 98.8)	43	76.4 (36.8; 94.3)
High	27	0.75 (0.61; 0.92)	29	82.4 (55.5; 94.3)	28	78.8 (41.4; 100.0)

Based on the interaction tests, the effect of cardiorespiratory fitness and muscular fitness on perceived work ability was different and it depended on the work demand occupation (blue collar or office workers). However, due to small number of office workers (around 20) the convergence was not achieved for all models and therefore the results according to the work demands were considered inclusive and thus not reported.

## Discussion

This cross-sectional study revealed that high cardiorespiratory fitness was associated with 67% lower sickness absence costs than those generated by low-fitness employees over the preceding 6 months. Moreover, work ability was statistically significantly higher among those with high or moderate cardiorespiratory fitness relative to the lowest tertile. These findings point to the potential for considerable cost savings at the societal level if sustained improvements in municipal workers’ cardiorespiratory fitness could be achieved.

The strength of the study lies in reliable accelerometer-measured information on physical activity and sedentary behaviour and register-based sickness absence days. A few limitations to this study’s design and implementation may have a bearing on the interpretation of the results. Firstly, given the cross-sectional nature of the study, no causal inference can be drawn. Another matter worth pointing out is that the data on sickness absence were collected for the 6 months before the measurement of physical activity, sedentary behaviour and cardiorespiratory fitness, whereas a prospective follow-up would be a more reasonable setting for ‘comparing like with like.’

The present results are consistent with those from prior studies pointing out the beneficial influence of cardiorespiratory fitness on sickness absence. Drake et al. found among office workers that high cardiorespiratory fitness decreased risk for all-cause sickness absence [8], while similar results found in nursing personnel with recurrent nonspecific low back pain [9]. However, while a positive influence of cardiorespiratory fitness was evident in our study, accelerometer-measured physical activity levels and meeting the physical activity recommendations did not show a statistically significant relationship with the number of sickness absence days, self-perceived work ability or quality of life. Prior work looking at questionnaire-reported vigorous physical activity has linked it to having fewer sickness absences [6], higher quality of life [27], and better work ability [10] or a stronger work ability index score [11]. Several factors could have accounted for divergent results. Firstly, the earlier work has relied mainly on self-reported questionnaire data on weekly mean amounts of physical activity during leisure time or on the way to work. This is in marked contrast to our study that employed accelerometer-measured physical activity and sedentary time. A device-based measurement is a major strength of our study since questionnaires are liable to overestimate or underreport physical activity or lack thereof [28]. However, due to the Hawthorne effect [29], it is possible that the use of accelerometer may have somewhat changed the participants’ physical activity behaviour. Secondly, different time horizons for sickness absence figures can produce divergent results. Also, our work benefited from the inclusion of not only the extended absences but also briefer sickness absences (≤10 working days). In most cases, information on short absences from work in Finland is accessible only through occupational healthcare providers and not publicly available.

According to Väisänen *et al.* [30] over the last two decades cardiorespiratory, fitness has declined in most occupational groups, particularly in blue-collar and low-skilled occupations. Consequently, procedures that encourage employees to improve good cardiorespiratory fitness, e.g., through commuting by walking or biking, climbing the stairs rather than use the elevator whenever possible might be effective approaches for improving cardiorespiratory fitness. Among manual labourers this goal might benefit from redesigning the work to promote individual work ability. A regular 6MWT-based evaluation of employees’ cardiorespiratory fitness might yield useful information for the prevention of sickness absences [6] and, thereby, improve health-related quality of life [27]. There are broader implications too; for instance, Hynninen *et al.* [13] identified a connection between perceived work ability and both future sickness absences and occupational healthcare costs.

In conclusion, reduced cardiorespiratory fitness is associated with substantial financial losses both for employers and society, arising from lost productivity manifested as sickness absences. The present findings suggest a link between high cardiorespiratory fitness with lower levels of all-cause sickness absences and also better work ability. Actions aimed at increasing employee physical activity may, accordingly, exhibit multiple positive consequences—for instance, lower costs arising from reduced sickness absences. Consequently, the importance of physical activity as a means to improve physical fitness should be further emphasized.
